# Novel resonant pressure sensor based on piezoresistive detection and symmetrical in-plane mode vibration

**DOI:** 10.1038/s41378-020-00207-0

**Published:** 2020-11-16

**Authors:** Xiangguang Han, Qi Mao, Libo Zhao, Xuejiao Li, Li Wang, Ping Yang, Dejiang Lu, Yonglu Wang, Xin Yan, Songli Wang, Nan Zhu, Zhuangde Jiang

**Affiliations:** 1 State Key Laboratory for Manufacturing Systems Engineering, International Joint Laboratory for Micro/Nano Manufacturing and Measurement Technologies, Xi’an Jiaotong University, 710049 Xi’an, China; 2 School of Mechanical Engineering, Xi’an Jiaotong University, 710049 Xi’an, China; 3 Xi’an Flight Automatic and Control Institute, 710049 Xi’an, China

**Keywords:** Engineering, Physics

## Abstract

In this paper, a novel resonant pressure sensor is developed based on electrostatic excitation and piezoresistive detection. The measured pressure applied to the diaphragm will cause the resonant frequency shift of the resonator. The working mode stress–frequency theory of a double-ended tuning fork with an enhanced coupling beam is proposed, which is compatible with the simulation and experiment. A unique piezoresistive detection method based on small axially deformed beams with a resonant status is proposed, and other adjacent mode outputs are easily shielded. According to the structure design, high-vacuum wafer-level packaging with different doping in the anodic bonding interface is fabricated to ensure the high quality of the resonator. The pressure sensor chip is fabricated by dry/wet etching, high-temperature silicon bonding, ion implantation, and wafer-level anodic bonding. The results show that the fabricated sensor has a measuring sensitivity of ~19 Hz/kPa and a nonlinearity of 0.02% full scale in the pressure range of 0–200 kPa at a full temperature range of −40 to 80 °C. The sensor also shows a good quality factor >25,000, which demonstrates the good vacuum performance. Thus, the feasibility of the design is a commendable solution for high-accuracy pressure measurements.

## Pressure sensor: Piezoresistive detection

The operating principle and experimental realization of a new piezoresistive resonant pressure sensor are shown. Resonant pressure sensors rely on changes in applied pressure to a diaphragm to cause the resonant frequency of the resonator to change. This enables high stability and signal-to-noise ratios, combined with a quasi-digital output; resultantly, resonant pressure sensors are often used in critical applications such as aerospace and for instrument calibration. Here, a team led by Zhuangde Jiang from Xi’an Jiaotong University, China, report a piezoresistive detection-based resonant pressure sensor. They establish the theory for their device operation, verified by both simulations and experiments. Their device shows an accuracy better than 0.02% FS and a resonant quality factor greater than 25,000.

## Introduction

Owing to their quasi-digital outputs with high stability and high signal-to-noise ratios (SNRs), resonant pressure sensors have higher accuracy and long-term stability than other kinds of pressure sensors, such as piezoresistive pressure and capacitive pressure sensors^1–4^. Resonant pressure sensors have been successfully utilized in the fields of aerospace, industry control, and instrument calibration and have been extensively researched in recent decades. The structures and different principles of silicon-based resonant pressure sensors and quartz-based resonant pressure sensors have been proposed along with the improvement in technologies for fabricating microelectromechanical systems (MEMSs)^5–11^. However, the availability of silicon resonator-based pressure sensors successfully developed for commercial applications remains limited.

Resonant pressure sensors based on capacitive detection methods have attracted considerable attention from researchers because they are easy to fabricate and have simple structures^4,6,12,13^. Ren et al.^13^ proposed a resonant pressure sensor on the basis of the principle of the capacitive detection method; the sensor showed a sensitivity of 8 Hz/kPa with a silicon on insulator wafer, and its total accuracy exceeded 0.05% full scale (FS), but the quality factor was small because a vacuum package was not used. Du et al.^5^ developed a resonant pressure sensor based on another capacitive detection with silicon fusion bonding and a silicon island amplifier; it had a good linearity of 0.02% FS and a sensitivity of 10 Hz/kPa. These capacitive detection-based resonant pressure sensors showed that the change in the detection capacitance was extremely small, requiring a signal amplifier circuit with an extremely large amplification factor; meanwhile, the crosstalk between an excitation comb and capacitance detection comb was hard to eliminate^12^. Electromagnetic detection-based resonant pressure sensors have been comprehensively researched by Li et al.^11^ and Lu et al.^14^; these sensors have a total accuracy that is >0.01% FS. However, external magnets must be mounted on the sensors, and thus the sensors have large package sizes and poor temperature sensitivity. Compared with capacitive and electromagnetic detection methods, the piezoresistive detection method has better SNR. Owing to the difficulties associated with fabrication, few researchers have exerted efforts on studying piezoresistive detection-based resonant pressure sensors; however, the DRUCK Company has proposed a piezoresistive detection-based resonant pressure sensor that has been commercialized^15^.

In the development of resonant pressure sensors, shielding signals from adjacent modes are important for close-loop control. If the frequency interval between a working mode and adjacent mode is insufficiently large and the output of the adjacent mode is insufficiently small, the working mode easily locks in the wrong mode during sensor operation. DRUCK^16^ proposed a novel structure that eliminates adjacent output signals, such as the first mode output; however, numerous beams are used, and thus the resulting structure is complex. Shi et al.^17^ proposed another beam combination structure by setting the piezoresistive detection beam at the end of the resonant beam, and the beam combination structures have good shielding ability for adjacent modes, but the resonator quality factor was only ~10,000 with vacuum packaging. Welham et al.^15^ proposed the use of two straight beams as coupling and detection beams for shielding the adjacent mode, and the increase in the stiffness owing to the coupling beam decreases the sensitivity. A 5-µm resonant beam was used to increase the sensitivity of the sensor, which was sufficiently narrow to induce large nonlinearity during resonant vibration. As an aspect of the sensor quality factor, nonlinearity should be achieved by employing a high-vacuum package, and among the reported resonant pressure sensors with high-quality factors, anodic bonding with getters is the most commonly used method^6,8^. However, until now, the anodic bonding interface of the reported resonant pressure sensor has only been adopted by one type of doping, boron doping or phosphorus doping, and whether anodic bonding with silicon interfaces composed of different doping could achieve a high-vacuum cavity has seldom been researched.

A piezoresistive high-accuracy resonant pressure sensor with a high-quality factor and without sensitivity to the adjacent mode is necessary.

In this paper, a novel piezoresistive detection-based resonant pressure sensor with in-plane double-ended tuning fork (DETF) design and electrostatic excitation is proposed. The pressure–frequency theory for enhanced DETFs with coupling structures is obtained and verified by simulations and experiments. A novel detection method with a low adjacent mode output signal is proposed for shielding the adjacent mode output. To achieve high-vacuum packaging, anodic bonding with different doped interfaces is utilized and validated. Experimental results show that the nonlinearity of the developed resonant pressure sensor is >0.02% FS after temperature and pressure compensation, and the resolution is only 5 Pa. The output quality factor of a resonator is >25,000 to satisfy the requirement of signal control.

## Material and methods

### Design of the resonant pressure sensor chip

**Fig. 1:**
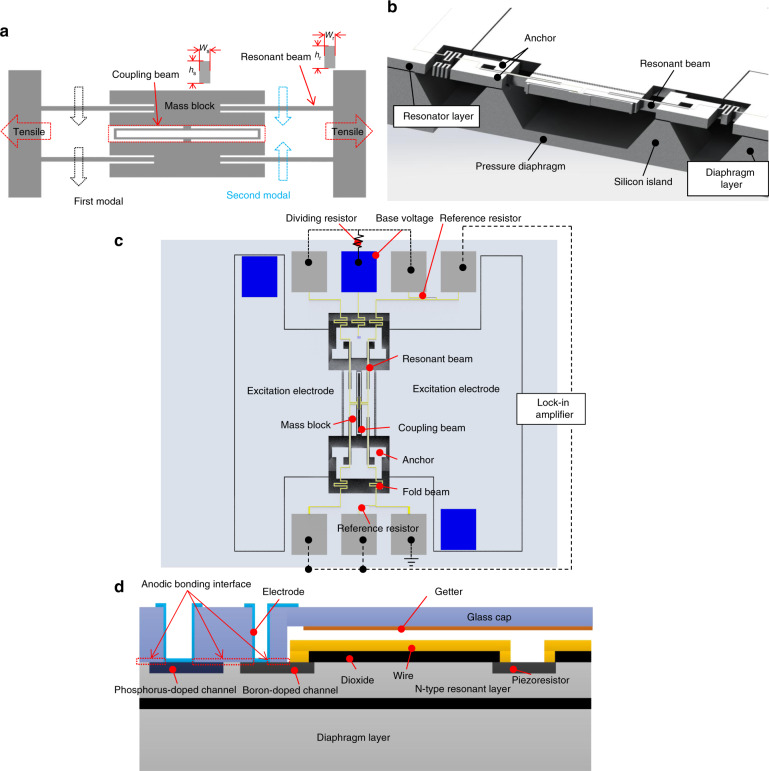
Overall design of the resonator pressure sensor. **a** Schematic of the resonator with a coupling beam-enhanced DETF, **b** cross-section view of the resonant pressure sensor chip, **c** detailed components of the resonator, and **d** vacuum package schematic by anodic bonding

As reported^12^, in the out-of-plane resonant mode, the vibration direction of the resonator is perpendicular to the diaphragm, resulting in substantial energy loss from the resonator to the diaphragm, which will decrease the *Q*-factor. Even for the in-plane common side mode, there is still a reaction force that is transferred to the diaphragm from the resonator due to the unbalanced dynamic structure; the quality factor will also be influenced by the diaphragm conditions, such as the width and thickness. The in-plane symmetry mode (second mode, shown as Fig. 1a) is a balanced mode with a low energy exchange between the resonator and pressure diaphragm since the vibration direction is parallel to the diaphragm. The symmetry vibration mode is generally obtained by using a coupling beam-enhanced DETF in resonator design, as shown in Fig. 1a. In recent years, the stress–frequency model for a resonator under the first resonant mode has been reported^18^; however, when the coupling beam is combined in the resonant structure, the second mode is usually selected as the working mode, and the relationship between the inner stress of the resonant beam and the working mode frequency needs to be updated. The working mode frequency is studied in part 1 of the Supplementary information and obtained as shown in Eq. (1):

1 $$f_2 = \frac{1}{{2\pi }}\sqrt {\frac{{\frac{{4{\it{Ew}}_{\mathrm{r}}^3{\it{h}}_{\mathrm{r}}}}{{{\it{l}}_{\mathrm{r}}^{3}}} \left( {{{1 + \sigma /\sigma }}_{{\mathrm{cr}}}} \right) + \frac{{4{\it{Ew}}_{\mathrm{s}}^3{\it{h}}_{\mathrm{s}}}}{{{\it{l}}_{\mathrm{s}}^{\it{3}}}}}}{{m_{\mathrm{mass}} + 0.3714m_{\mathrm{r}} + 0.3714m_{\mathrm{s}}}}}$$

where *σ* is the inner axial stress of the resonant beam, *σ*_cr_ is the buckling stress of the resonant beam, *w*_r_, *l*_r_, and *h*_r_ are the width, length, and height of the resonant beam, respectively, and *w*_s_, *l*_s_, and *h*_*i*_ are the width, length, and height of the coupling beam, respectively. The equivalent mass of the resonator is expressed as *m*_E_ = *m*_mass_ + 0.3714*m*_r_ + 0.3714 *m*_s_^19^, where *m*_mass_ is the mass of the mass block, *m*_r_ is the mass of the resonator beam, and *m*_s_ is the mass of the coupling beam.

The overall design of the proposed resonant pressure sensor chip adopts “glass–silicon–silicon” structure layers. The cross-sectional view of the entire main structure is shown in Fig. 1b. The top glass layer serves as a high-vacuum package layer with a vacuum cavity, the middle silicon layer is the resonator layer with a thickness of 80 µm (N-type-100), and the bottom layer is the pressure diaphragm layer (N-type-100). The core component is the resonator, which is bonded on the silicon island by the fusion bonding method. The measured pressure is applied to the diaphragm, then the anchors move toward the outside through the silicon island on the diaphragm, and the resonator beam is stretched to the tensile state. The frequency of the resonator changes accordingly. The sensor chip is designed with an absolute pressure range of 0–200 kPa and a sensitivity of 19 Hz/kPa. Its second original resonant frequency is ~54 kHz. The resonant pressure sensor chip adopts electrostatic comb excitation, which potentially involves a linear driving force. All the detailed components of the resonator are shown in Fig. 1c.

For the electrical connection, as shown in Fig. 1c, the excitation electrodes (fixed and movable combs) are linked to the external circuit by the phosphorus doping area (blue regions), whereas detection pads are doped by boron (gray regions). The detection circuit (base voltage) will be isolated by the PN junction to the excitation circuit at the doping pad. When the bodies of the movable comb and mass block (N-type) have a higher electrical level by 1–5 V compared with the boron-doping area (P-type), and since the PN junction is reversed within the working temperature of −40 to 85 °C, the current leakage is so small that it can be ignored. For the sake of isolation between the silicon and signal wire on the beams, the silicon surface is covered by 300 nm SiO_2_, except for the doping and bonding areas. The signal wires pass through the fold beam to the external circuit.

A high-vacuum package is very important for high-quality factors. Based on experience, in the anodic bonding process, the maximum roughness for the anodic bonding interface should be <20 nm for the vacuum package requirement, and thus metal wire passing through the bonding interface has difficulty achieving high-vacuum packaging. To lead out the signal of the resonator, the “boron-doped channel” and “phosphorus-doped channel” are introduced as the “detection channel” and “excitation channel” between the resonator and electrodes on the glass in Fig. 1d, and then the usage of metal wire is avoided. The overall bonding area is composed of a phosphorus-doped area, boron-doped area, and original N-type silicon, and the minimum bonding width is 200 μm. Aluminum wires are then fabricated on the glass surface and glass holes and used to connect the resonator layer to the external circuit. The getter is sputtered on the glass to maintain vacuum stability. For the detection circuit, the vibration signal will be detected by a Wheatstone bridge, with two detection resistors on the coupling beam and two reference resistors on the resonator side, with each piezoresistor value of ~5.5 kΩ.

### Piezoresistive detection method

**Fig. 2:**
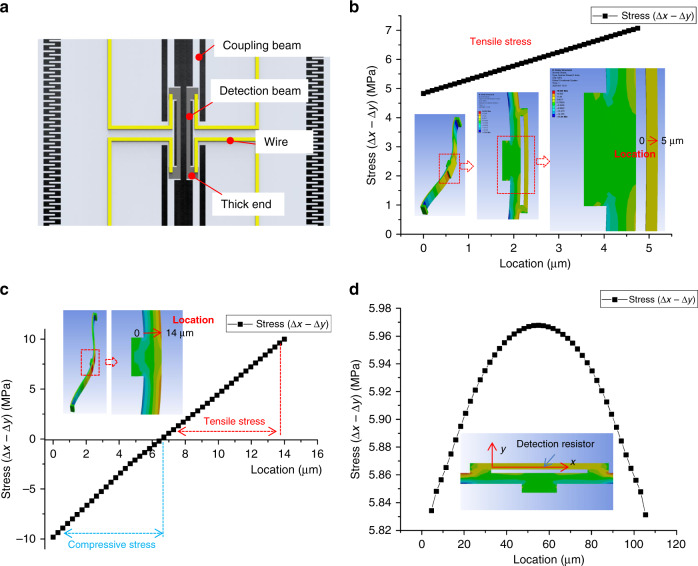
Detection structure design. **a** Design of the piezoresistive detection structure, **b** stress distribution of the coupling beam with the detection beam, **c** stress distribution of the coupling beam in a resonant state of normal coupling, and **d** stress distribution along the detection beam

Piezoresistive resistors are widely used in MEMS piezoresistive sensors^20,21^, and in resonant pressure sensors, piezoresistor layouts are important for resonant pressure sensor signal detection quality, such as the adjacent mode signal shielding ability and vibration amplitude sensitivity. Based on the resonant mode simulation and static pressure simulation results, the coupling beam will not be deformed during first-order mode vibration; thus, placing the detection piezoresistors in the coupling beam to decrease noise from adjacent modes is reasonable. However, the stress distribution of the crossing coupling beam is obviously complex, as shown in Fig. 2c. The stress status changes from compressive stress to tensile stress crossing the coupling beam width. If detection piezoresistors are directly fabricated on the coupling beam, the piezoresistor location along the coupling beam width direction fluctuates with the mask alignment tolerance (between the piezoresistor mask and resonator DRIE (Deep Reactive Ion Etching) mask); thus, the stress of the piezoresistor is uncertain.

To solve this issue, a novel piezoresistive detection method is proposed (Fig. 2a). Two short detection beams are designed apart from the coupling beams. The detection piezoresistor is connected to the coupling beam with “thick-end” structures on both sides. In the simulation results in Fig. 2b, d, when the detection beam is added, the stress of the detection beam becomes pure tensile stress or compressive stress during resonator vibration. Meanwhile, since the stress decreases as the detection beam width increases, a detection beam width of 5 µm is selected in consideration of the fabrication ability, and the average stress difference (Δ*x* − Δ*y*) of the piezoresistor on the detection beam is ~5.92 MPa with a 0.2 μm resonant amplitude, which satisfies the requirement of piezoresistive signal detection.

### Mode analysis

For the prevention of mode interference, the desired balanced mode must be completely separated from the adjacent modes over the entire working frequency range^15^. The front six mode responses of the proposed resonant pressure sensor is analyzed using ANSYS, as shown in Supplementary Fig. S2. The working mode of the resonator is the second mode, with a basic frequency of ~53.152 kHz. The intervals between the working and adjacent modes (first and third modes) are all >20% of the original frequency and can thus satisfy the close-loop control. There is no frequency intersection between the working mode and adjacent modes within the full pressure range.

## Results and discussion

### Micromachining process

**Fig. 3:**
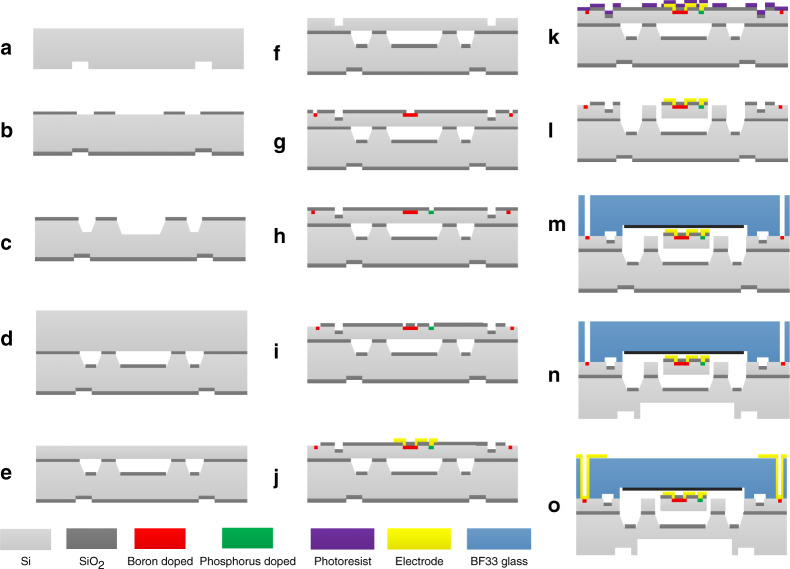
Fabrication process of the resonant pressure sensor chip. **a** Making back marks with RIE; **b** patterning the SiO_2_ for wet etching; **c** wet etching of the silicon island with a depth of 250 μm; the etching time is controlled; **d** high-temperature fusion silicon bonding; **e** thinning and polishing the resonator layer to the desired height; **f** making front mark with RIE; **g** B doping for the piezoresistor and electrode pad; **h** P doping for the electrode pad of the excitation electrode; **i** opening the window of the SiO_2_ for anodic bonding and the wire; **j** fabrication of the wire by the lift-off process; **k** patterning the photoresist for the DRIE; **l** DRIE to fabricate the resonator; **m** high-vacuum anodic bonding; **n** DRIE releasing the pressure sensing diaphragm; and **o** sputtering electrode upon the glass surface

The resonant pressure sensor chip is fabricated using MEMS technology based on 4-in. (N-100) single crystal silicon wafers with a thickness of 400 µm. The flow chart of fabrication is shown in Fig. 3. Wet etching is used in fabricating the silicon island with tetraethyl ammonium hydroxide and a 500-nm thermal oxidation SiO_2_ mask. Silicon fusion bonding is then adopted for the bonding of the resonator layer to the diaphragm layer. When lapping and chemical–mechanical polishing of the resonant layer is finished, the detection piezoresistors, reference piezoresistors, and boron-doping connectors are fabricated by ion implantation. For good ohmic contact with the excitation electrode, P-ion implantation is performed after B-ion implantation. The lift-off process is used in aluminum wire fabrication. Then, DRIE is used to release the overall structure of the resonator. The glass cap with a tapered hole and getter is then bonded on the resonator layer through anodic bonding technology at a temperature of 400 °C and voltage of 900 V. The pressure diaphragm is then obtained by DRIE. Finally, the electrodes are fabricated with sputtering technology for electronic connection.

**Fig. 4:**
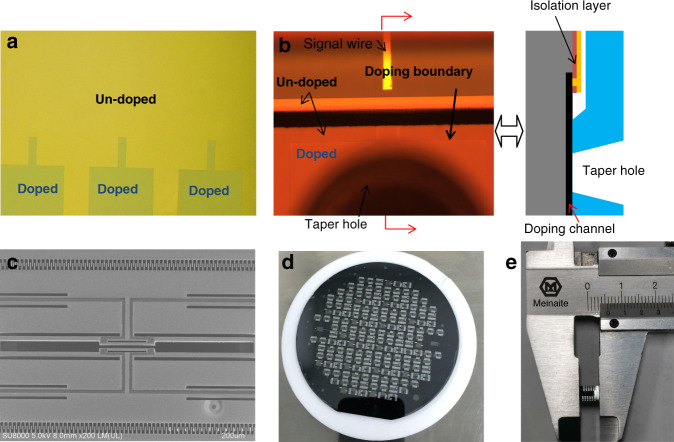
Fabricated sensor chip. **a** Chip surface after heavy doping and mask SiO_2_ removed, **b** view through the glass after anodic bonding, **c** SEM of the fabricated resonator, **d** wafer level of anodic bonding after electrode fabrication, and **e** the chip size shown after dicing

For the different doped anodic bonds, the bonding surface is wished as planar as possible to obtain a high-vacuum package. In the different heavy doping processes, SiO_2_ is always used as the doping mask, and inductively coupled plasma (ICP) etching is usually selected as the patterning method due to its high-dimensional control accuracy compared with wet etching. However, during the ICP process, it is inevitable that the silicon under the mask for the heavy-doped window will be slightly etched by a few tens of nanometers, which is hard to control and may decrease the vacuum sealing quality. The overetching of the heavy doping area is shown in Fig. 4a, and the depth is measured by a step profiler at ~30–60 nm among different wafers. To validate whether the electrostatic force during the anodic bonding is large enough to pull the gap together, the simulation is performed, and the results show that the gap can be easily collapsed when the voltage is >200 V with a glass thickness of 400 μm. The top view of the bonded interface through the glass is shown in Fig. 4b, and good bonding quality is shown for the different doping areas, although the doping boundary can still be distinguished.

The fabricated resonant sensor chips are shown in Fig. 4c. All the key dimensions of the resonator are precisely fabricated within a tolerance of 0.5 μm. The wafer-level anodic high-vacuum bonding is good, as shown in Fig. 4d. The dimension of the resonant pressure sensor chip is only 4.7 mm × 5.7 mm, which is much smaller than the reported resonant pressure sensors^6,14^, in Fig. 4e.

### Performance characteristics

**Fig. 5:**
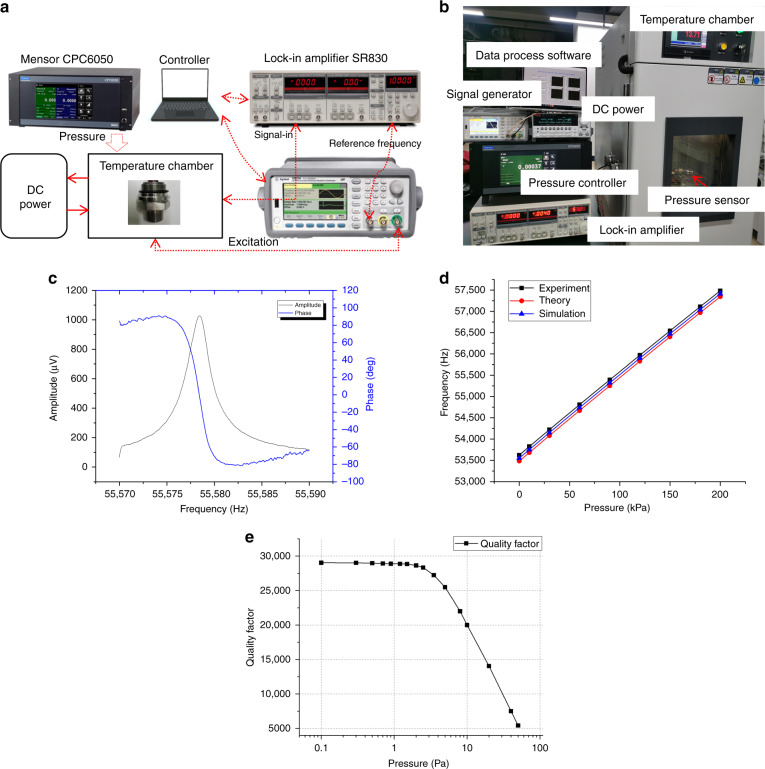
Experimental platform and the resonant pressure sensor test results. **a** Schematic of the open-loop test platform for the resonant pressure sensor, **b** experimental system for the resonant pressure sensor, **c** resonant pressure sensor response at atmosphere pressure, **d** frequency output compared with the theoretical result and simulation result, and **e** the quality factor versus the cavity pressure

The performance of the fabricated resonant pressure sensor is verified. An Agilent function generator 33500b is used to generate an excitation signal for the resonator. A lock-in amplifier SR830 is utilized to obtain the frequency response in an open-loop scanning manner. A Mensor CPC6050 precision pressure controller is used to generate standard pressure with a high reading precision within ±0.01% FS, and an environment test chamber FWD701P with an accuracy of ±0.3 °C is used. The open-loop test platform is shown in Fig. 5a, and the real setup is shown in Fig. 5b.

As shown in Fig. 5c, the preliminary measurement results show that the fabricated resonant pressure sensor has a working frequency of ~55,798 Hz at a temperature of 20 °C and atmospheric pressure. The quality factor can be calculated with the value of 26,756. For the vacuum performance of the cavity validation, the quality factor versus the pressure experiment is tested. As Fig. 5e shows, the quality factor will be largely decreased when the vacuum is >5 Pa, and the quality factor will increase smoothly when the vacuum pressure decreases from 5 to 0.1 Pa. Since the quality factor reaches 26,756, it is estimated that the vacuum of the cavity is <2 Pa according to the *Q*-factor pressure curve, which indicates that the vacuum package works well. Meanwhile, there is an ~180° phase jump around the resonant frequency point in Fig. 5c. The voltage output of the resonator is ~1000 μV with the Wheatstone bridge supply voltage of 2 V, so there will be no need for a greater amplifier circuit for signal detection.

The relationship between the resonant frequency and the pressure (0–200 kPa) is measured at room temperature to validate the performance of the pressure sensor. The fabricated resonant sensor has a sensitivity of ~19 Hz/kPa. The overall frequency shift of the full pressure range is ~3.8 kHz. The results of the numerical calculation, simulation, and experiment are shown in Fig. 5d. The numerical result can be calculated by Eq. (1). The axial stress is simulated by ANSYS because obtaining it through a numerical calculation is difficult due to the complex route from the diaphragm to the resonant beam. The experimental results are in good agreement with the analytical and numerical results at the full pressure range. The maximum error between the theoretical results and experiment results is <3%. The theoretical model can be useful in guiding the design of the resonant pressure sensor with an enhanced DETF structure.

**Fig. 6:**
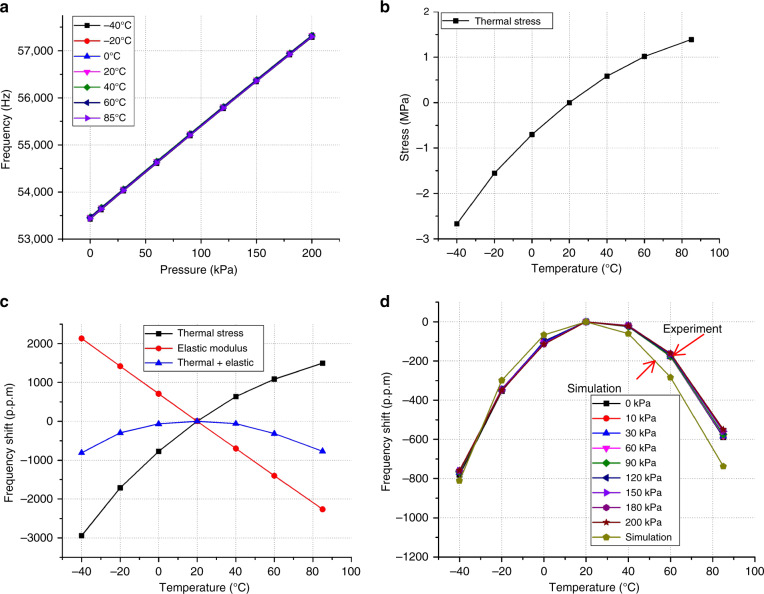
Temperature influence research of the resonant pressure sensor. **a** Frequency response under a full pressure range (0–200 kPa) and temperature range (−40 to +85 °C), **b** thermal stress of the resonant beam under different temperatures, **c** frequency shift caused separately by the thermal stress and TCE, and frequency shift influenced by the both factors, and **d** the experimental results of the frequency shift compared with the simulation results

The frequency under the full pressure range (0–200 kPa) and full temperature range (−40 to +85 °C) is tested, and the uncompensated result is shown as Fig. 6a. Correspondingly, the temperature frequency shift, when the frequency is set at 20 °C as the reference point, is calculated at different applied static pressure levels, as shown in Fig. 6d. It is obvious that the frequency shift curves do not change linearly when the temperature rises, but show high consistency between all the curves. The temperature coefficient of frequency (TCF) of the resonator is calculated as follows:

2 $${\mathrm{TCF = }}\frac{{{f}_1 - {f}_{20\,^\circ{\mathrm{C}}}}}{{{f}_{20\,^\circ {\mathrm{C}}} \left( {{T}_1 - {T}_{20\,^\circ {\mathrm{C}}}} \right)}}$$

where *f*_1_ is the resonator frequency under the applied temperature and *f*_20 °C_ is the frequency at 20 °C. For TCF generation, the silicon-based resonator TCF is almost determined by the thermal stress and the TCE (temperature coefficient of elasticity) of silicon^22^. The TCF caused by the TCE is ~−30 p.p.m./°C^23^, while in this design, tensile thermal stress will be generated when the temperature is rising since the CTE of BF33 is larger than that of silicon. The thermal stress is simulated by ANSYS, and the thermal stress is not completely linear since CTE_Silicon_ increases with increasing temperature, while CTE_BF33_ changes little^22,24^. The simulated thermal stress is shown in Fig. 6b. Then, taking the thermal stress into Eq. (1), the mean TCF caused by thermal stress is ~+30 p.p.m./°C, which can almost cover the TCF caused by TCE, and then the TCF total will be obtained as shown in Fig. 6c, which largely decreases by the counteraction. From the comparison result in Fig. 6d, the experimental results are in good agreement with the simulation results; the nonlinearity of the frequency temperature curve can be attributed to the increase in CTE_Silicon_. It can be seen that the TCF within the temperature 0 to +40 °C is extremely small (TCF_max_ = 5 p.p.m./°C and TCF_min_ = −2 p.p.m./°C), which is much lower than the silicon resonator TCF value of −30 p.p.m./°C, but TCF increases rapidly when the temperature is *T* > 40 °C or T < 0 °C (TCF_max_ = 13 p.p.m./°C at −40 °C, TCF_min_ = −10 p.p.m./°C at 85 °C).

**Fig. 7:**
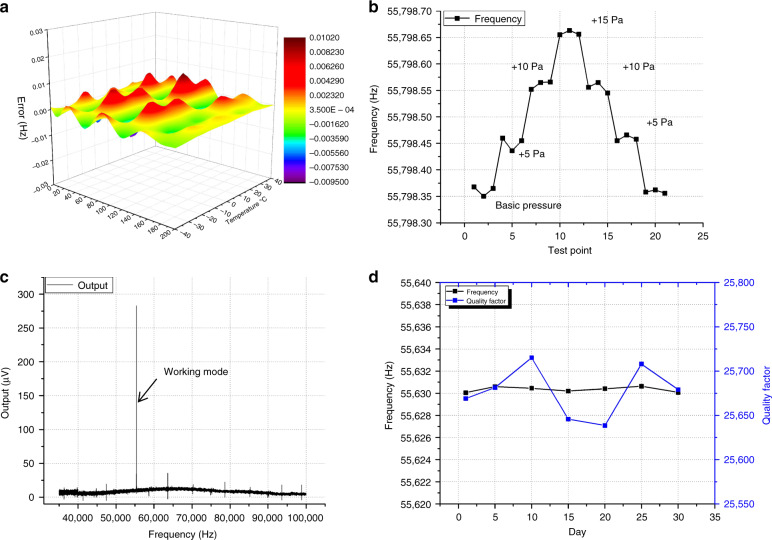
Resonant pressure sensor performance test. **a** Nonlinearity response surface within a full pressure range and temperature range, **b** resolution ability test results, **c** output shielding of adjacent mode validation, and **d** the long-term stability test under a pressure of 100 kPa and 20 °C

After polynomial compensation of pressure and temperature, the nonlinearities are <0.02% FS at a full pressure range and full temperature range, as shown in Fig. 7a. Efforts have been made to validate the resolution ability with an open-loop measurement setup. The test is performed with a basic pressure of 100 kPa and a temperature of 20 °C, and the test is performed three times at each pressure point. It can be seen that 5 Pa can be identified with the fluctuation of each pressure point of ~2 Pa, as shown in Fig. 7b.

To validate the shielding performance of the detection design, the output of adjacent modes is tested by open-loop frequency sweeping from 35,000 to 100,000 Hz, which covers the first and third modes, as shown in Fig. 7c, and the result shows that only the second mode can be detected, which demonstrates the good shielding ability of the detection structure.

The long-term stability of the resonant pressure sensor is researched after aging treatment, as shown in Fig. 7d. The results show that the fluctuation of the long-term stability test is ~±0.2 Hz, the test is performed with a stable pressure of 100 kPa and a stable temperature of 20 °C. However, since the temperature in the chamber during different days has a slight difference due to its limited accuracy (±0.3 °C), the frequency output will be influenced slightly. Meanwhile, the pressure source will also have an influence on the fluctuation since the pressure controller accuracy is ~±0.01% FS. These results indicate that the fabricated resonant pressure sensor has good long-term stability.

The comparison of the sensor performance between this paper and typical reported resonant pressure sensors in recent years is shown in Table 1. Compared with other studies, this paper has proposed a novel piezoresistive detected single resonator-based pressure sensor that combines almost all key parameters (quality factor, sensitivity, temperature range, and nonlinearity) with high performance.

**Table 1:** Performance comparison

	*Q*-factor	Sensitivity (Hz/kPa)	Nonlinearity (%)	Temperature	Resonator type	Resonator number
Sun et al.^12^	10,000	29	0.02	−40 to +80 °C	Capacitance	Single
Du et al.^6^	22,795	20	0.21	+30 to +75 °C	Capacitance	Single
Yan et al.^25^	16,000	11.89	0.01	−45 to +65 °C	Piezoresistance	Double
Zhang et al.^26^	–	36.58	0.014	−20 to +60 °C	Quartz	Triple
This paper	27,650	19	0.02	−40 to 85 °C	Piezoresistive	Single

## Conclusion

This paper presents a stress–frequency numerical model for a resonator pressure sensor with an enhanced DETF structure and proposes the design and fabrication of a piezoresistive detection-based resonant pressure sensor. A novel detection method is proposed with the ability to shield adjacent mode signals. For the preparation of a high-vacuum package, the boron- and phosphorus-doped channels in the anodic bonding interface are used to connect the resonator and external circuit. The experimental results show that the compensated nonlinearity of the sensor is within 0.02% FS, its quality factor is >25,000 in a pressure range of 0–200 kPa, and at a full temperature range of −40 to +85 °C. Our future works will focus on closed-loop circuit design for portable application and optimizing the package stress release for improving long-term stability. During the current fabrication, the depth–width ratio is ~26:1, which will result in a large machine cost, so the resonant layer and the minimum line width for etching will be optimized in future work.

## Supplementary information

Supplemental Material
