# Supplementary material for: Novel resonant pressure sensor based on piezoresistive detection and symmetrical in-plane mode vibration
Source: Microsyst Nanoeng. 2020 Nov 16;6:95. doi: 10.1038/s41378-020-00207-0 (PMC8433135; doi:10.1038/s41378-020-00207-0)
Supplement: Supplementary file 1 — Supplemental Material [file 41378_2020_207_MOESM1_ESM.docx]

Novel Resonant Pressure Sensor Based on Piezoresistive Detection and Symmetrical In-plane Mode Vibration

**supplementary material**

**1. Stress-frequency numerical Model for enhanced DETF**

The symmetry vibration mode is generally obtained by using a coupling beam-enhanced DETF in resonator design. This type of DETF decreases anchor loss. As Chen reported [19], the stress–frequency model for a resonator under the first resonant mode has been studied, which can be expressed as **Eq. S(1)**. However, when the coupling beam is combined in the resonant structure, the second mode is selected as the working mode, and **Eq.S (1)** becomes unsuitable for output analysis and must be updated.

| $f_{1}=f_{10}\sqrt{1+\sigma/\sigma_{cr}}$ | (1) |
| --- | --- |

where *f*_1_ is the frequency of resonator under stress, *f*_10_ is the first order original frequency of the resonator, *σ* is the inner axial stress of the resonant beam, and *σ_cr_* is the buckling strength of the resonant beam and can be obtained as follows [21]:

| $\sigma_{cr}=\frac{P_{cr}}{A}=\frac{\pi^{2}EI}{ul^{2}A}$ | (2) |
| --- | --- |

where *P_cr_* is buckling load of the resonant beam, *E* is the Young’s modulus of silicon, *I* is the moment of inertia of the resonant beam, *μ* is the length factor of resonant beam, *l* is the length of resonant beam, and *A* is the cross area of a resonant beam.

In a resonator with enhanced DETF, the first order frequency *f*_1_ with pressure can be expressed as **Eq. S(3)**, where *k_r_* is the stiffness of the resonant beam under pressure, *m_E_*_1_ is the equivalent mass of the resonator and expressed as *m_E_*_1_*=m_mass_+*0.3714*m_r_+m_s_* [20], where *m_mass_* is the mass of the mass block, *m_r_* is the mass of resonator beams, and *m_s_* is the mass of coupling beam.

The original *f*_10_ in **Eq. S(1)** can be expressed as **Eq. S(4)**, where *w_r_* is the width of resonant beam, *h_r_* is the height of resonant beam, *l_r_* is the length of resonant beam, and *k_r_*_0_ is the original stiffness of the resonant beam.

| $f_{1}=\frac{1}{2\pi}\sqrt{\frac{k_{r}}{m_{\text{E1}}}}$ | (3) |
| --- | --- |
| $f_{10}=\frac{1}{2\pi}\sqrt{\frac{k_{r0}}{m_{E1}}}=\frac{1}{2\pi}\sqrt{\frac{4Ew_{r}^{3}h_{r}/l_{r}^{3}}{m_{E1}}}$ | (4) |

By integrating **Eq. S(3)** and **Eq. S(4)** into **Eq. S(1)**, the equation can be transformed as follows:

| $k_{r}=\frac{4Ew_{r}^{3}h_{r}}{l_{r}^{3}}\cdot\left(1+\sigma/\sigma_{cr}\right)$ | (5) |
| --- | --- |

The second order frequency of the resonator under applied pressure can be expressed as follows when the coupling beam is considered:

| $f_{2}=\frac{1}{2\pi}\sqrt{\frac{K_{\text{E}}}{m_{\text{E2}}}}=\frac{1}{2\pi}\sqrt{\frac{k_{r}+k_{s}}{m_{\text{E2}}}}$ | (6) |
| --- | --- |

Where *K_E_* is the equivalent stiffness of resonator of the second order mode, including the stiffness *k_r_* of resonant beam and stiffness *k_s_* of coupling beam, *k_s_=Ew_s_*^3^*h_s_/l_s_*^3^, *w_s_* , *h_s_* and *l_s_* is the width, thickness and length of coupling beam, and *m_E2_=m_mass_+*0.3714*m_r_+*0.3714*m_s_*.

When the measured pressure is applied to the diaphragm anchored to the resonator in the sensor chip, tensile stress is generated only in the resonator beam, and thus *k_s_* is a constant. By taking **Eq. S(5)** into **Eq. S(6)**, the relationship between the inner stress of the resonant beam and the working mode frequency can be obtained through **Eq. S(7)**.

| $f_{2}=\frac{1}{2\pi}\sqrt{\frac{\frac{4Ew_{r}^{3}h_{r}}{l_{r}^{3}}\cdot\left(1+\sigma/\sigma_{cr}\right)+\frac{4Ew_{s}^{3}h_{s}}{l_{s}^{3}}}{m_{mass}+0.3714m_{r}+0.3714m_{s}}}$ | (7) |
| --- | --- |

The key dimensional parameters of the resonator used in the simulation are shown as **Table S1**.

For the study of the influence of the key dimensional parameters on frequency–stress sensitivity and the basic frequency value, resonant beam width *w_r_* and coupling beam width *w_s_* are researched. Numerical analysis results obtained by Matlab software are shown in **Figure S1a**. The second order mode frequency of the resonator increases with *w_r_*. In the frequency–stress sensitivity curve, a turning point (*w_r_* = 24 µm) has the largest value of 67.5 Hz/MPa in the widening process of resonator beam width, in Figure 1a. In subsequent studies, the width of the resonator beam will be set at 24 µm.

When the resonant beams and mass block are fixed, the frequency–stress sensitivity of the resonator decreases and initial frequency increases with the width of the coupling beam increasing, as the numerical results shown in **Figure S1b**. As experience, the frequency stability of the resonator decreases with narrower vibration beam width (resonant and coupling beams). In our design, the width of the coupling beam is set as 12 µm.


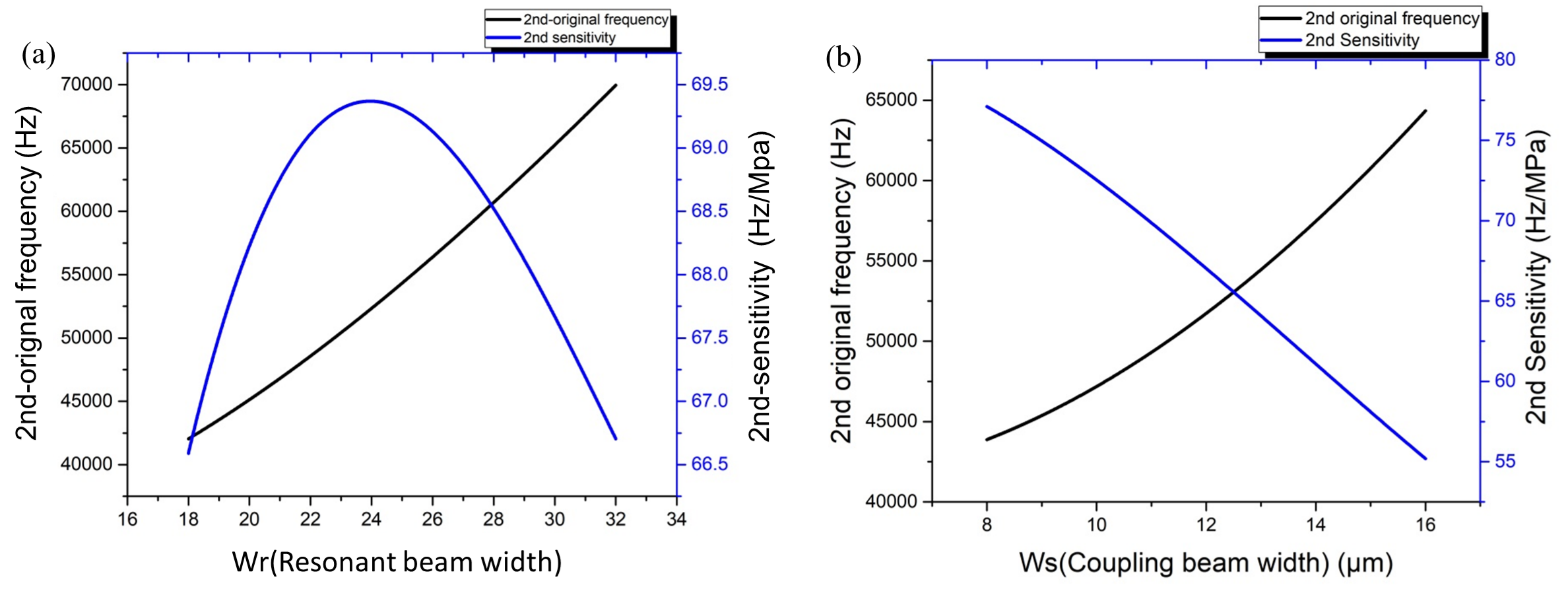


**Figure S1** Different width of resonant beam and coupling beam influence research, **a** frequency-stress sensitivity and second order original frequency with different resonant beam width, **b** frequency-stress sensitivity and second order original frequency with different coupling beam width

**Table S1.** Structure parameters of resonant pressure sensor chip

| Parameters | Value |
| --- | --- |
| Resonant beam | 25 μm×550 μm×80 μm |
| Coupling beam | 14 μm×370 μm×80 μm |
| Detection beam | 5 μm×120 μm×80 μm |
| Silicon island height | 250 μm |
| Comb size | 3 μm×20 μm |
| Comb gap | 3 μm |
| Fold beam width | 14 μm |
| Diaphragm thickness | 80 μm |
| Resonator thickness | 80 μm |
| Glass thickness | 400 μm |
| Overall size of sensor chip | 4.7 mm×5.7 mm |

**2. Simulation**

2.1 Mode simulation of front six modes under different stress

**
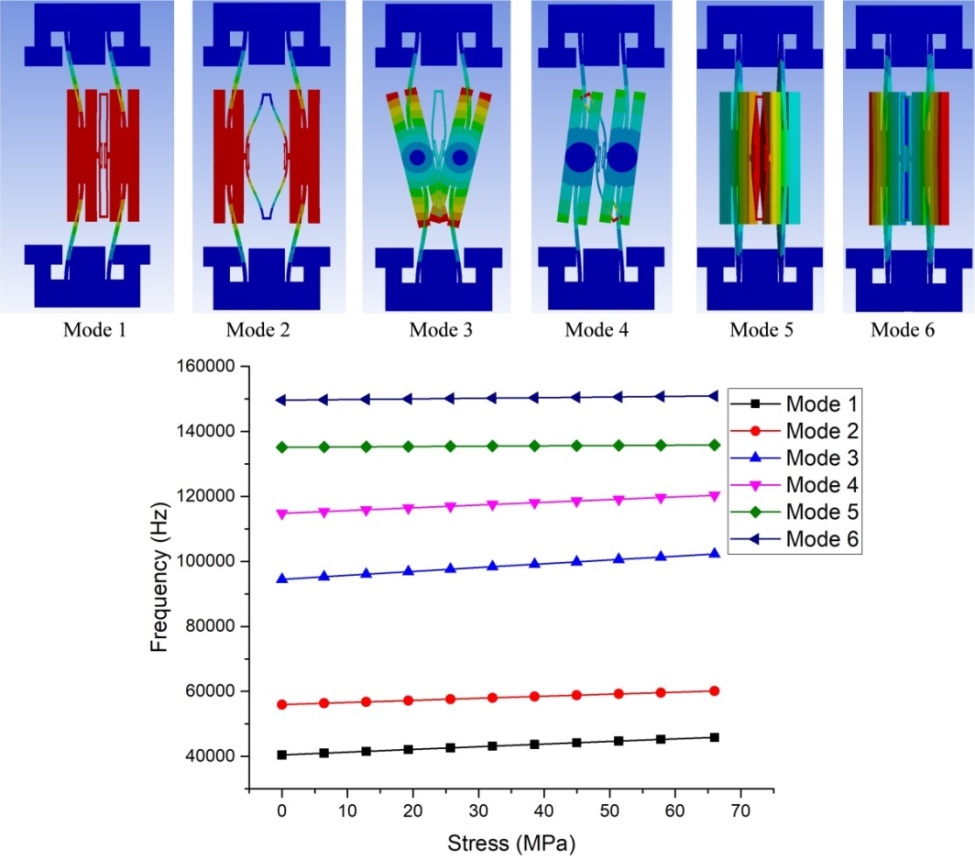
**

**Figure S2** The front six modes simulation result under different stress

2.2 Static structure analysis under full pressure

The static structure analysis with the pressure of 200 kPa is analyzed by ANSYS, and the axial stress along the resonant beam is generated with an average value of 60 MPa, as shown in **Figure S3**.


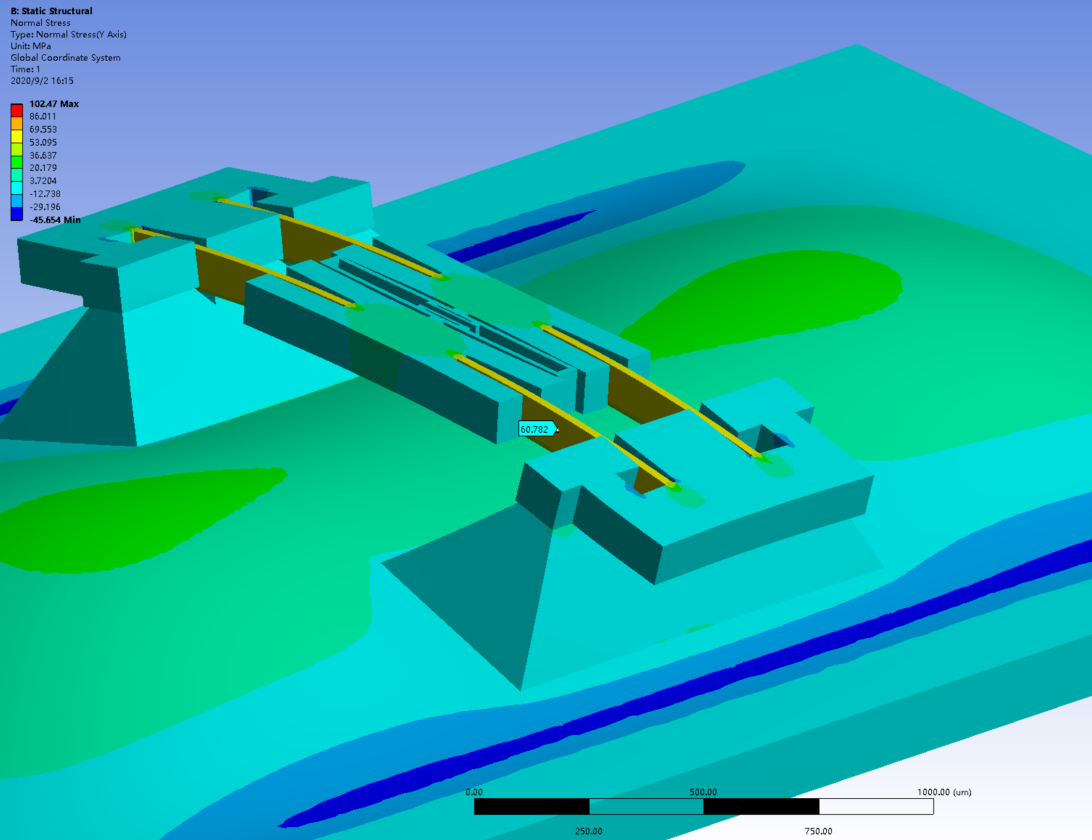


**Figure S3** Static structure analysis of the resonant beam under full pressure
